# Supplementary material for: The contribution of tourism mobility to tourism economic growth in China
Source: PLoS One. 2022 Oct 27;17(10):e0275605. doi: 10.1371/journal.pone.0275605 (PMC9612534; doi:10.1371/journal.pone.0275605)
Supplement: S1 Fig — (DOCX) [file pone.0275605.s001.docx]

**Supporting information**

The study area is divided into seven regions according to geographical divisions of China. North China including Beijing, Tianjin, Hebei, Shanxi, Inner Mongolia. Northeast including Heilongjiang, Jilin, Liaoning. East China including Shanghai, Jiangsu, Zhejiang, Anhui, Jiangxi, Shandong, Fujian. Central China including Henan, Hubei, Hunan. South China including Guangdong, Guangxi, Hainan. Southwest China including Chongqing, Sichuan, Guizhou, Yunnan, Tibet. Northwest China including Shaanxi, Gansu, Qinghai, Ningxia, Xinjiang. Figure S1 shows a map of the seven regions. The map base in this study comes from the "Standard Map Service of the Ministry of Natural Resources of China"(http://bzdt.ch.mnr.gov.cn/). The drawing review number of all maps in this study is GS (2016) 1599.


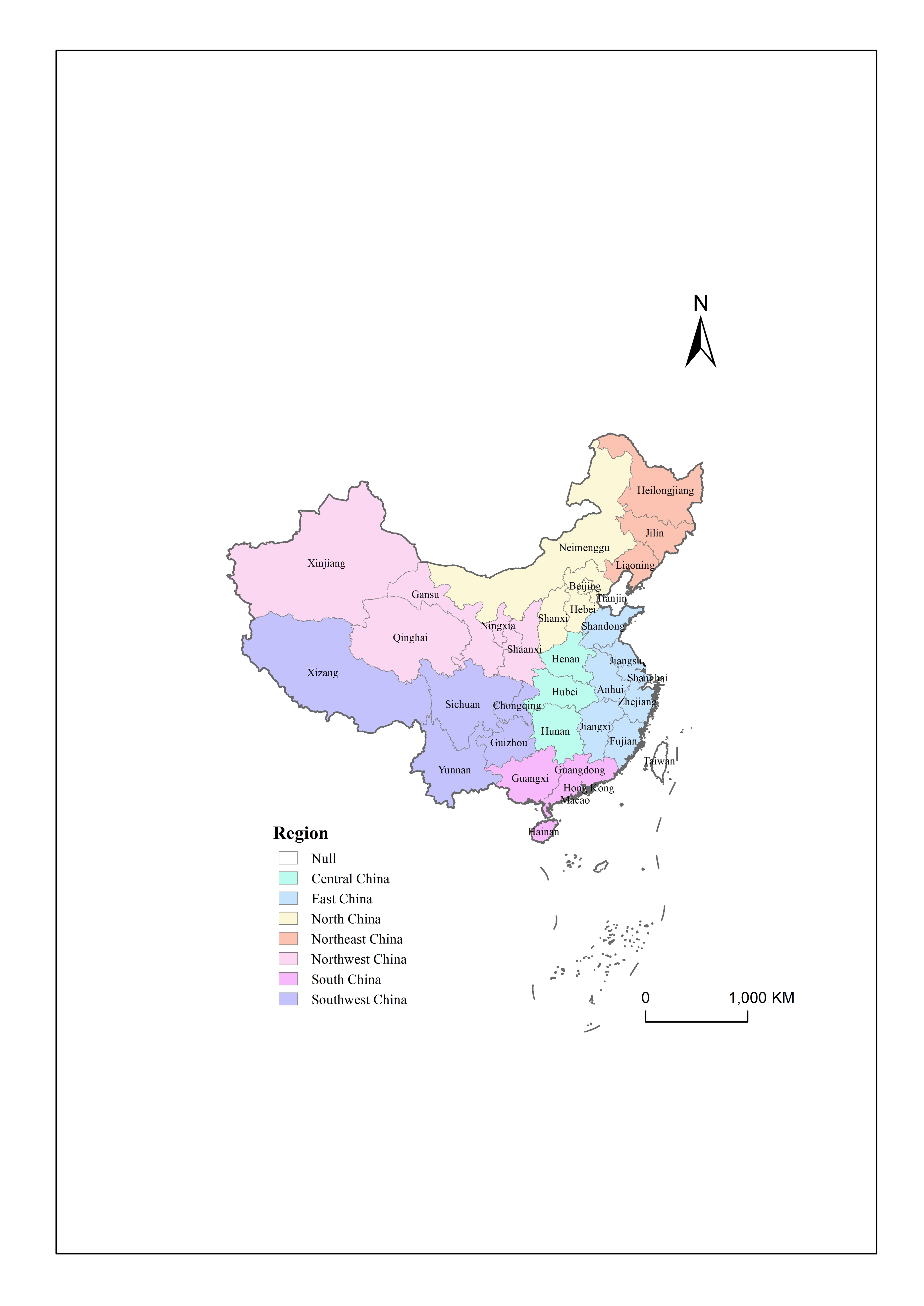


**Fig.S1 Map of the seven regions.**
