# Supplementary material for: The contribution of tourism mobility to tourism economic growth in China
Source: PLoS One. 2022 Oct 27;17(10):e0275605. doi: 10.1371/journal.pone.0275605 (PMC9612534; doi:10.1371/journal.pone.0275605)
Supplement: Attachment — Submitted filename: 20220523_Review_Plos_One_01.docx [file pone.0275605.s003.docx]

**Review of “The Contribution of Tourism Mobility to Tourism Economic Growth in China”**

The manuscript has the potential to make various contributions to the tourism-related policy literature but should be strengthened by addressing several critical deficiencies (outlined below).

(a) Introduction

- It would be helpful to briefly explain the connections between tourism mobility (TM) and tourism economic growth (TEG) from the geospatial perspective early in the paper and return to this when discussing the policy implications of the research findings.
- Although the authors have given some explanation of the current situation of economic development in China, it is currently insufficient. More context is needed regarding the present situation of the tourism industry, TM, and TEG.
- The research question(s) and main hypothesis or hypotheses should be included in this section.
- The second and third paragraphs of this section should be restructured as a new, separate literature review section. The authors are encouraged to include more previous studies and discuss them in greater depth in the literature review, as the literature reviewed in the original version of the manuscript is insufficient.

(b) Econometric model

- A few words or a sentence should be sufficient to describe the spatial weight matrix used in equation 4.

(c) Data sources

- The last paragraph of this section (about the seven regions of the study area) should be moved to the Appendix. Including a map inset of these areas would be helpful here, as well.

(d) Spatial evolution

- It would be helpful to include a LISA cluster map of to help readers better understand the spatial patterns described here.

(e) Discussion

- This section does not sufficiently analyze the authors’ findings in the context of previous studies. How and why does this study differ from previous research? Relatedly, there are some similarities between this study’s results and those of previous research—what is the unique contribution of this paper? Please emphasize these differences more clearly.
- It seems that the policy implications of this manuscript are limited. Thus, the authors are encouraged to derive policy implications or recommendations based on their findings. Such implications need not be restricted to Chinese tourism-related policies but may also be applicable to other countries with conditions similar to China. The authors are encouraged to clarify these issues in this section.
- Theoretical and practical contributions should be highlighted more explicitly in this section.
- The authors are encouraged to expand their discussion of the research limitation(s), as this issue is currently insufficiently explained.

**Final comments**

Given the several critical deficiencies pointed out above, this paper is not ready for publication in its current form. I recommend a major revision, with resubmission possible if the authors are able to logically and satisfactorily address these deficiencies.
